# REAL EXPERIENCE OF FRONT-LINE NURSING HOME STAFF DURING COVID-19: A SYSTEMATIC REVIEW AND META-SYNTHESIS

**DOI:** 10.1093/geroni/igad104.3554

**Published:** 2023-12-21

**Authors:** Xiufen Yang, Chaoran Qu, Weisi Peng, Lile Xiong, Hua Guan, Weixiang Luo, Yiping Cheng

**Affiliations:** Shenzhen People’s Hospital, Shenzhen, Guangdong, China (People’s Republic); Shenzhen People’s Hospital, Shenzhen, Guangdong, China (People’s Republic); Shenzhen People’s Hospital, Shenzhen, Guangdong, China (People’s Republic); Shenzhen People’s Hospital, Shenzhen, Guangdong, China (People’s Republic); Shenzhen People’s Hospital, Shenzhen, Guangdong, China (People’s Republic); Shenzhen People’s Hospital, Shenzhen, Guangdong, China (People’s Republic); Shenzhen Hospital of Southern Medical University, Shenzhen, Guangdong, China (People’s Republic)

## Abstract

**Objective:**

The COVID-19 has become less of a threat around the world. However, the threat to nursing homes and older adult still exists. The study aimed to explore the challenges and coping strategies of front-line nursing home staff during COVID-19 and provide recommendations for coping strategies and measures for public health of emergency in nursing homes in future. Design: Qualitative Meta-synthesis. Date sources: From the beginning until 30 May 2023, searches were conducted in five international databases (CINAHL, PubMed, Web of Science, PsycINFO and Cochrane library). The studies were screened and reviewed separately by two reviewers using the checklist from Joanna Briggs Instifor assessing qualitative research. A pragmatic meta-aggregator approach was applied to synthesise the findings.

**Results:**

The meta-synthesis included 16 qualitative studies and four mixed studies, which included 642 participants were analyzed to identify 199 findings that were organized into 15 categories and combined into three syntheses. Three synthesised findings were concluded: ①The challenge of the front-line nursing home staff during the COVID-19, ②The coping strategies of the front-line nursing home staff during the COVID-19, and ③Suggestions for future nursing homes to deal with public health of emergency. Conclusion(s): Social/Health organizations and government, especially health organization, should make joint efforts to provide guidance knowledge and practice, and long-term material and mental support for nursing homes. To improve the quality of life of residents and staffs in nursing homes, further research should implement high-quality interventions based on the victory fighting epidemic experience and clinical treatment.

